# Beware of beam damage under reaction conditions: X-ray induced photochemical reduction of supported VO_*x*_ catalysts during *in situ* XAS experiments†

**DOI:** 10.1039/d2cp02721f

**Published:** 2022-08-25

**Authors:** Anna Zabilska, Adam H. Clark, Davide Ferri, Maarten Nachtegaal, Oliver Kröcher, Olga V. Safonova

**Affiliations:** Paul Scherrer Institute 5232 Villigen Switzerland olga.safonova@psi.ch; École Polytechnique Fédérale de Lausanne 1015 Lausanne Switzerland

## Abstract

*In situ* X-ray absorption spectroscopy (XAS) is a powerful technique for the investigation of heterogeneous catalysts and electrocatalysts. The obtained XAS spectra are usually interpreted from the point of view of the investigated chemical processes, thereby sometimes omitting the fact that intense X-ray irradiation may induce additional transformations in metal speciation and, thus, in the corresponding XAS spectra. In this work, we report on X-ray induced photochemical reduction of vanadium in supported vanadia (VO_*x*_) catalysts under reaction conditions, detected at a synchrotron beamline. While this process was not observed in an inert atmosphere and in the presence of water vapor, it occurred at room temperature in the presence of a reducing agent (ethanol or hydrogen) alone or mixed with oxygen. Temperature programmed experiments have shown that X-ray induced reduction of VO_*x*_ species appeared very clear at 30–100 °C but was not detected at higher temperatures, where the thermocatalytic ethanol oxidative hydrogenation (ODH) takes place. Similar to other studies on X-ray induced effects, we suggest approaches, which can help to mitigate vanadium photoreduction, including defocusing of the X-ray beam and attenuation of the X-ray beam intensity by filters. To recognize beam damage under *in situ*/*operando* conditions, we suggest performing X-ray beam switching (on and off) tests at different beam intensities under *in situ* conditions.

## Abstract 2

X-ray photoreduction of V^5+^ in supported vanadia catalysts was detected by XAS during *in situ* experiments at a synchrotron beamline. Practical strategies helping to recognize and mitigate such problems are suggested.

## Graphical abstract

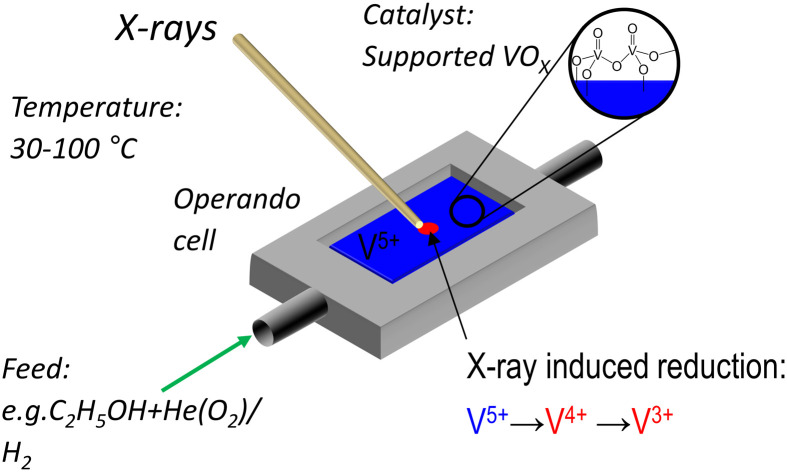

## Introduction

Spectroscopic methods applied under *in situ* and/or *operando* conditions are powerful tools for understanding the mechanisms of catalytic reactions including the structure of the catalytic active site.^1,2^ X-Ray absorption spectroscopy (XAS) is a particularly powerful tool in this respect because it is element-specific and provides information on the electronic and local geometric structure of metal species.^3,4^ In recent decades, research on catalytic mechanisms has benefited from the development of time-resolved energy-dispersive XAS and quick XAS methods and their proliferation across numerous light sources.^5–10^ Fast XAS acquisition allows the *in situ* (*operando*) experiments to be performed under transient conditions that allows for the detection of active sites directly involved in redox reactions.^11–15^ While performing *in situ*/*operando* investigations, one typically expects to observe only chemically induced transformations; however, an additional reactive component, the X-ray beam, should not be neglected.

X-Ray induced damage is a common issue in the X-ray crystallography of proteins^16–19^ and XAS of biological samples and samples in water.^20–24^ In such systems, X-ray irradiation may lead to the formation of radicals and electrons, which can cause sample damage including reduction of metal species.^16–24^ Inorganic water-free systems in this regard are more stable; however, several cases of photo-reduction were reported in X-ray photoelectron spectroscopy (XPS),^25,26^ where beam damage was enhanced by the use of vacuum. Radiation damage observed during XAS experiments on heterogeneous catalysts has rarely been reported. Newton *et al.*^27^ investigated a Cu(ii)-containing zeolite in heterogeneously catalyzed methane oxidation to methanol and found that Cu(ii) may undergo reduction in the presence of methane or pure helium upon X-ray irradiation. Albrahim *et al.*^28^ investigated a highly dispersed RhO_*x*_/Al_2_O_3_ catalyst by *in situ* XAS and detected that rhodium clusters undergo reduction even at room temperature in the presence of hydrogen and intense X-rays; *in situ* heating to 310 °C leads to further agglomeration of clusters, comparable to agglomeration typically observed at 650 °C without exposure to the X-ray beam. Van Schooneveld and DeBeer^29^ showed that iron in K_3_[Fe(CN)_6_] and manganese in KMnO_4_ can undergo X-ray-induced reduction during XAS measurements; the authors investigated the relationship between the X-ray dose and the extent of metal reduction by measuring these samples at different synchrotron beamlines.

Vanadium-based catalysts are among the most common redox-active systems used in homogeneous and heterogeneous catalyses.^30,31^ The mechanisms of heterogeneous catalytic reactions of selective oxidation and reduction over vanadium-based catalysts are still being revised by modern *in situ* and *operando* X-ray-based techniques, such as XPS and XAS.^32–40^ There are several publications, which describe X-ray-induced vanadium reduction during XPS experiments.^26,41–43^ It was suggested that the electron–hole pair generation or Auger decay may lead to holes in the valence band, which from a physical point of view are similar to bond breaking.^41^ Vanadium reduction in these publications was associated with the loss of lattice oxygen, which is enhanced by ultra-high vacuum,^43^ whereas the presence of low levels of oxygen in the gas phase prevented or slowed down this process.^42^

In our previous work, using *operando* quick XAS,^36^ we showed that the rate of ethanol oxidation to acetaldehyde over a bilayered 5 wt% V_2_O_5_/15 wt% TiO_2_/SiO_2_ catalyst (consisting of sub-monolayer VO_*x*_ species anchored onto a TiO_*x*_ monolayer supported on SiO_2_) is kinetically coupled to the V^5+^ → V^4+^ redox process. The effect of the X-ray beam on the kinetic data at the catalysis relevant temperatures (above 150 °C) was tested and excluded. We did, however, detect unexpected vanadium reduction below 100 °C, which we associate with X-ray irradiation. This warrants a detailed study of beam-induced vanadium reduction, the results of which are presented here. To understand the nature and the conditions of vanadium photoreduction, we performed a series of experiments showing that below 100 °C the X-ray beam does not affect vanadium speciation in air and in the presence of oxygen, helium, or water vapor. Only a combination of the X-ray beam with a reducing agent, such as ethanol, hydrogen, or an ethanol–oxygen mixture, resulted in the photochemical reduction of vanadium. Herein, we systematically study the X-ray-induced reduction of vanadium and propose approaches to recognize and suppress it.

## Materials and methods

The majority of experiments described in this work were performed using a model bilayered 5 wt% V_2_O_5_/15 wt% TiO_2_/SiO_2_ catalyst,^44^ which we used in our previous work which was focused on the redox activity of vanadium and titanium during thermocatalytic ODH of ethanol.^36^ This catalyst has close to one monolayer titanium coverage and was prepared as an analog of a widely used V_2_O_5_/TiO_2_ catalyst. The latter one demonstrates outstanding activity in a variety of redox catalytic reactions,^45–48^ however, it could not be efficiently investigated by time-resolved V K-edge XAS due to almost full X-ray absorption by titanium (the Ti K-edge, 4966 eV, is located just below the V K-edge, 5465 eV). Thus, a 5 wt% V_2_O_5_/15 wt% TiO_2_/SiO_2_ catalyst was chosen as a compromise: on one hand, its titania loading is sufficient to promote the activity of vanadium species,^36,44^ and on the other hand, it still allows measuring V K-edge XAS with a time-resolution of 1–10 s.^36^

### Materials

The detailed catalyst synthesis and characterization procedures of the 5 wt%V_2_O_5_/15 wt%TiO_2_/SiO_2_ catalyst can be found elsewhere.^36,44^ The 5 wt% V_2_O_5_/15 wt% TiO_2_/SiO_2_ catalyst has a surface density of *ca.* 4.9 Ti nm^−2^, which corresponds to monolayer TiO_*x*_ dispersion on silica.^49^ The VO_*x*_ surface density is equal to 1.8 V nm^−2^, which is below that of monolayer dispersion (8 V nm^−2 ^^50^). Vanadium surface species are present as dimeric and trimeric VO_*x*_ species; no crystalline V_2_O_5_ was detected by Raman spectroscopy.^44^

For comparison, we also investigated a titania-free 8 wt% V_2_O_5_/SiO_2_ catalyst, which contains *ca.* 2.3 V nm^−2^ mostly present as isolated tetrahedral VO_4_ species.^51^

### V K-edge XAS experiments

#### XAS measurements

XAS investigations were performed at the SuperXAS beamline, Swiss Light Source (SLS), Villigen, Switzerland. The SLS operated at 2.4 GeV and a ring current of 400 mA. The polychromatic beam coming from the 2.9 T superbend magnet was first collimated by a Si-coated mirror (which also served to reduce higher harmonics), then monochromatized by a Si(111) channel-cut crystal monochromator, and subsequently focused by a Rh-coated toroidal mirror. The typical beam spot size at the sample position measured 200 × 400 μm^2^ (unless otherwise stated) and the total flux was about 1.2 × 10^11^ ph s^−1^. Scanning of the X-ray energy around the absorption edge was done by oscillation of the Si(111) channel-cut monochromator with a frequency of 1 Hz (2 scans per s). V K-edge XAS spectra of the catalyst were recorded in the fluorescence mode^52^ by using a PIPS diode (Mirion Technologies) as a detector. Energy calibration was performed through the measurement of a vanadium foil (V K-edge at 5465 eV) in the transmission mode at the beginning of each experiment by moving the *operando* cell away from the X-ray beam. The incident and the transmitted beam intensities were measured using 15 cm long ionization chambers filled with 0.5 bar N_2_ and 0.5 bar He. The V K-edge XAS data were processed using the in-house developed ProQEXAFS software.^53^ Spectra recorded from the 5 wt% V_2_O_5_/15 wt% TiO_2_/SiO_2_ catalyst were averaged every 20 scans and those from the 8 wt% V_2_O_5_/SiO_2_ catalyst every 10 scans. For background subtraction and normalization, linear functions were used.

#### Operando cell

For *in situ*/*operando* XAS experiments, we used a stainless steel plug-flow reactor (cell), described in detail elsewhere.^54^ The cell is equipped with a graphite window (thickness 0.25 mm, Fisher Scientific), partially transparent for X-rays. The sample (*ca.* 15 mg, 63–150 μm fraction) was placed between two quartz wool plugs. The heating of the cell was done with two parallel-connected heating cartridges (Moesch), whereas the temperature was controlled by a thermocouple placed inside the reactor. Three gas lines were connected to the cell (typically oxygen, ethanol, and pure helium); two of them were equipped with three-way switching solenoid valves (Parker, Series 9) allowing fast gas switching. This cell allows fast gas exchange; 95% of the gas is replaced within 2.4 s at a total flow of 50 mL min^−1^ (Fig. S5, ESI†).

The gas flows were controlled using mass-flow controllers (Bronkhorst). To provide ethanol flow, the helium flow (purity 99.997%, 30 mL min^−1^) passed through a saturator filled with ethanol (purity 99.997%) and kept at 8 °C. The concentration of oxygen was varied by admixing the initial oxygen source (14 or 40 vol% O_2_ in He) with helium. The total flow passing through the cell was constant in every experiment and was equal to 50 mL min^−1^.

Before every experiment (unless stated otherwise), the catalyst was pre-treated inside the cell by heating in a flow of 14 vol% O_2_ in He to 400 °C at 12 °C min^−1^ and dwelling for 30 min.

#### Ethanol–oxygen temperature-programmed experiment (TPE)

The collection of V K-edge XAS was started at 30 °C in oxygen (6.4 vol% O_2_ in He). After one min, the feed was switched to 1.6 vol% EtOH, 6.4 vol% O_2_ in He. One min later, heating was started with a heating rate of 5° min^−1^.

#### Ethanol TPE

Recording of V K-edge XAS spectra was started at 30 °C. After one min, the oxygen flow was replaced by 1.6 vol% EtOH. One minute later, heating was started with a rate of 5° min^−1^.

#### Ethanol-feeding experiment

The V K-edge XAS measurement started in oxygen (14 vol% in He); after one min, the oxygen feed was replaced by 1.6 vol% EtOH in He.

#### X-Ray beam switch-off experiment

The catalyst was pre-treated and cooled down in 6.4 vol% O_2_ in He to 160 °C and then the feed was switched to the catalytic mixture containing 1.6 vol% EtOH, 6.4 vol% O_2_ in He. The ethanol ODH was performed in a low conversion regime until steady-state conditions (controlled by product analysis) were reached (*ca.* 30 min). Afterward, the reaction temperature could be further adjusted between 50 and 160 °C. V K-edge XAS recording was started under steady-state conditions at the desired temperature. After 10 min, the X-ray beam was switched off by closing the shutter (while not stopping the data acquisition). After 10 min, the shutter was opened again and V K-edge XAS spectra were recorded for another 10 min. In this experiment, the X-ray beam size at the sample position measured 500 × 400 μm^2^.

#### Experiments with reduced beam intensity

A series of experiments was carried out with a reduced X-ray beam intensity, which was achieved by inserting Al-filters of 20, 40, 60, and 80 μm thickness, which resulted in a reduction of the incident beam intensity to 46, 21, 10, and 5% of the full beam intensity, respectively (details in the ESI,† Section 1.2). Before the experiments, the catalyst was pre-treated in 6.4 vol% O_2_ in He, cooled down to 50 °C, and subsequently exposed to the ethanol–oxygen feed (1.6 vol% EtOH, 6.4 vol% O_2_ in He) for 20 min in the absence of the X-ray beam. Recording of the V K-edge XAS was started with the use of the thickest Al-filter (80 μm, 5% of incident beam intensity), after which the filter thickness was gradually decreased. With each filter thickness, XAS spectra were collected for 10 min. At the end of the experiment, XAS spectra of the V K-edge foil were recorded and used for energy calibration. In this experiment, the X-ray beam size at the sample position measured 500 × 400 μm^2^.

#### V K-edge XAS analysis

To follow the transformation of vanadium in *in situ*/*operando* experiments, the pre-edge intensity and the half-height edge position descriptors were determined for every spectrum with the use of a bespoke MatLab program. To calculate the area under the pre-edge peak and the pre-edge center of mass position, first, the rising edge was fitted with the use of a cumulative distribution function and subtracted from the spectrum. The resulting peak was integrated (details are in the ESI,† Section 1.3).

### Product analysis

During *operando* investigations, the gas composition at the outlet of the reactor cell was analyzed using a Fourier transform infrared (FT-IR) spectrometer (Bruker Alpha II) equipped with a 70 mm path length cell (95% of the gas is replaced in 15 s) heated to 150 °C to prevent condensation. The absorbance (47 scans per spectrum) was recorded in the 4000–500 cm^−1^ range at a resolution of 4 cm^−1^. This allowed a time resolution of the product gas analysis of 1 min, which was particularly important during temperature-programmed experiments. Before every experiment, the background spectrum was recorded in an oxygen flow. To quantify the products, specific spectral regions corresponding to the selected molecules were extracted and analyzed using the multivariate curve resolution alternating least square (MCR-ALS) analysis^55^ (Fig. S4, ESI†). For quantitative calibration, the spectra of pure compounds with different concentrations were included in the experimental set for the MCR-ALS analysis.

## Results and discussion

### The identification of X-ray beam-induced vanadium reduction

Oxidative dehydrogenation of ethanol over supported VO_*x*_ catalysts proceeds *via* the Mars–van Krevelen mechanism.^45,56,57^ The step of ethanol oxidation by lattice oxygen, which is accompanied by oxygen vacancy formation and metal reduction, is followed by the step of catalyst re-oxidation by molecular oxygen. Mechanistic investigations showed that the step of catalyst re-oxidation by oxygen is faster than the step of catalyst reduction by ethanol. As a result, in the presence of oxygen in the gas feed, the majority of vanadium species are in the highest V^5+^ oxidation state.^36,45,56,58,59^ In our previous work,^36^ using *operando* quick XAS under steady-state and transient conditions, we quantitatively showed that the rate of ethanol oxidation over a 5 wt%V_2_O_5_/15 wt%TiO_2_/SiO_2_ catalyst above 150 °C is kinetically coupled to the V^5+^ to V^4+^ reduction process. The effect of the X-ray beam on the kinetic data at the catalytic relevant temperatures (above 150 °C) was tested by varying the beam density and based on these tests excluded. Nevertheless, at temperatures below the catalysis relevant conditions, we observed previously unreported beam effects that we focus on in detail in the present work.

**Fig. 1:**
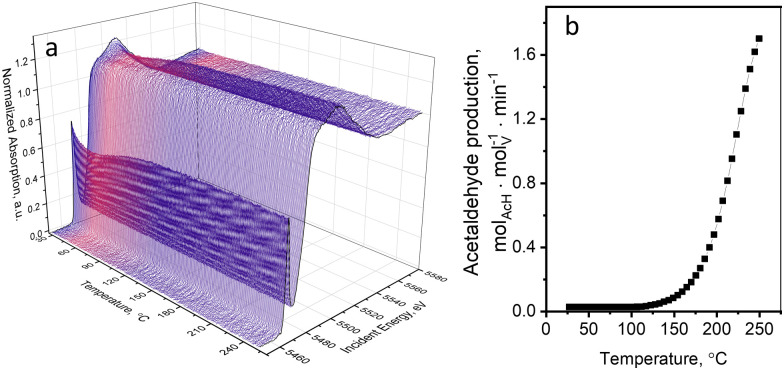
(a) V K-edge XANES spectra of the 5 wt% V_2_O_5_/15 wt% TiO_2_/SiO_2_ catalyst during TPE in ethanol–oxygen feed (1.6 vol% EtOH, 6.4 vol% O_2_ in He); and (b) the rate of acetaldehyde production detected by FTIR spectroscopy in the same experiment.

Fig. 1a shows the evolution of V K-edge XANES spectra of a 5 wt%V_2_O_5_/15 wt%TiO_2_/SiO_2_ catalyst during an *operando* temperature-programmed experiment (TPE) in ethanol–oxygen feed, where the fully oxidized catalyst was exposed to an ethanol–oxygen flow (1.6 vol% EtOH, 6.4 vol% O_2_ in He) while heating from 30 to 250 °C. Fig. 1b shows the rate of acetaldehyde formation detected at the outlet of the cell, which confirmed that the catalyst becomes active at around 120 °C. Unexpected changes in the V K-edge XAS spectra appeared below the temperature of ethanol oxidation, at 30–100 °C (Fig. 1a). In this temperature interval, the pre-edge peak intensity decreased and the V K-edge edge position shifted towards lower energies. At higher temperatures, when the catalyst became active, the spectra were almost reverting to the initial state.

The pre-edge peak in V K-edge XAS spectra is observed due to the dipole-forbidden 1s–3d electron transition, which becomes partially allowed in non-centrosymmetric structures due to 3p–4d orbital mixing and overlap of the V 3d with ligand (O) 2p orbitals.^60,61^ The pre-edge shape and position depend on the structural parameters such as geometry around vanadium, length of bonds, oxidation state, and type of ligands.^61,62^ Characteristics of the pre-edge (such as position, height, and intensity) are often used to identify and semi-quantify the oxidation state and geometry of vanadium in unknown compounds.^61,63–67^ The edge position is another important spectral feature often used in the literature for the determination of the oxidation state.^61,66–69^ In our previous work, we measured V K-edge spectra of 21 reference compounds with known structure, containing vanadium atoms in different oxidation states and surrounded by oxygen in different local geometries, which are potentially helpful to identify the oxidation state of unknown vanadium structures.^36^ We showed that using the edge position and the pre-edge surface area (or pre-edge height) one can estimate the oxidation state and the coordination number of vanadium in an unknown state. The edge position and the pre-edge intensity (height and area) generally increase in the order V^3+^ < V^4+^ < V^5+^. The pre-edge intensity, besides, depends on the coordination number of vanadium and decreases in the following order: tetrahedral–pentahedra–octahedra (for details refer to ref. 36 and to the ESI,† Section 3.1). In this work, for the rapid qualitative analysis of the oxidation state of vanadium, we decided to use two descriptors: the pre-edge height and the edge position.

**Fig. 2:**
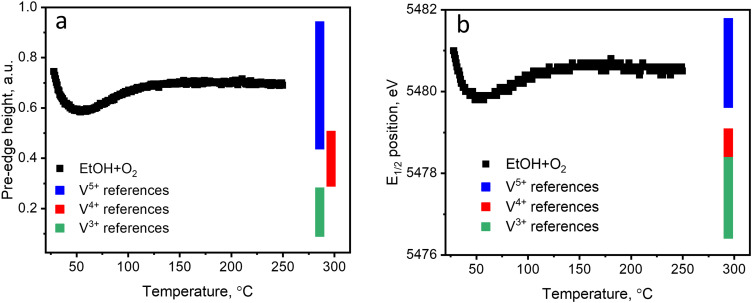
(a) The pre-edge height and (b) the half-edge step position of the references (indicated by the colored bars) and of the catalyst measured during TPE in ethanol–oxygen feed (1.6 vol% EtOH, 6.4 vol% O_2_ in He); details are in Fig. S12 (ESI†).

Fig. 2 (black curves) shows the changes in the pre-edge height and the edge position of each XAS spectrum during ethanol–oxygen TPE. The color bars indicate the ranges of values of the descriptors corresponding to vanadium references in V^3+^, V^4+^, and V^5+^ oxidation states (details in Fig. S12, ESI†). At temperatures relevant to ODH of ethanol, *i.e.* at 110–250 °C, the pre-edge height and the edge position trends for the catalyst show that vanadium species are mostly in the +5 oxidation state. As expected, during ethanol ODH vanadium re-oxidation is faster than vanadium reduction, thus, in the ethanol–oxygen feed, the resting oxidation state of vanadium in VO_*x*_ species is close to +5. However, at low temperatures, when acetaldehyde is not detected in the outlet (30–110 °C, Fig. 1b), the pre-edge intensity is lower and the position of the edge shifts towards lower energies. These changes may suggest that vanadium undergoes partial reduction. However, based on the values of the descriptors, which overlap for V^5+^ and V^4+^ references (especially for the pre-edge height), these experiments could not exclude that the observed spectral changes are due to an increase in the coordination number of VO_*x*_ species (see also Fig. S12, ESI†), *e.g.* due to the coordination of ethanol.

**Fig. 3:**
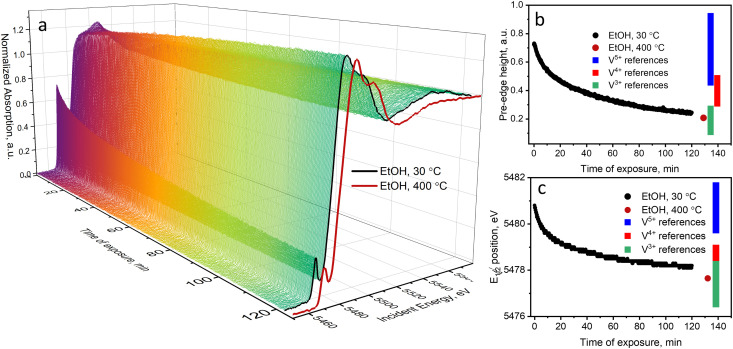
(a) V K-edge XANES spectra of the 5 wt% V_2_O_5_/15 wt% TiO_2_/SiO_2_ catalyst and (b) time profiles of the pre-edge intensity and (c) the edge position measured during the ethanol-feeding experiment at 30 °C (1.6 vol% EtOH in He). With dark red color, we show (a) the V K-edge spectrum of the catalyst reduced at 400 °C in ethanol (1.6 vol% EtOH in He) as well as (b) its pre-edge height and (c) half-edge step position.

To further test the transformations taking place at low temperatures, we exposed the catalyst to an oxygen-free ethanol-containing feed (1.6 vol% EtOH in He) at 30 °C for two hours. The V K-edge spectra measured in this experiment and the corresponding changes in the V pre-edge height and the edge position are shown in Fig. 3. After two hours of exposure of the catalyst to the X-ray beam, the pre-edge intensity decreased from 0.74 to 0.25 a.u. in the normalized V K-edge XAS spectra, whereas the edge position shifted from 5481 to 5478 eV. Both descriptors suggest strong vanadium reduction, which proceeds beyond V^4+^, towards V^3+^. For comparison, the V K-edge spectrum of the catalyst reduced at 400 °C in ethanol (1.6 vol% in He) is also shown in Fig. 3 (dark red curve in (a) and red dot in (b) and (c)). Under these conditions, vanadium is mostly in the +3 oxidation state.^36^ Interestingly, vanadium reduction of supported VO_*x*_ species by alcohol (methanol, ethanol) at room temperature was previously reported for VO_*x*_/TiO_2_ catalysts using *in situ* ambient pressure XPS^34,35^ and for VO_*x*_/SiO_2_ using high-resolution V K-edge XANES (using the K_β5,2_ emission line).^70^ In these studies, the authors assumed that vanadium reduction must be related to ethanol desorption, which, however, disturbs the electron balance.

To verify whether the reduction of vanadium in our catalyst by ethanol occurs at low temperatures, we used *in situ* diffuse reflectance (DR) UV-Vis spectroscopy, which is a complementary method sensitive to vanadium oxidation state changes in supported VO_*x*_ catalysts.^44^ The experimental conditions were very similar to those of the XAS (see the ESI,† for figures and additional discussion, Section 3.2). DR UV-Vis spectroscopy detected no significant vanadium reduction in the ethanol-feeding experiment at 50 °C, which suggests that vanadium reduction detected by XAS is X-ray beam-induced.

**Fig. 4:**
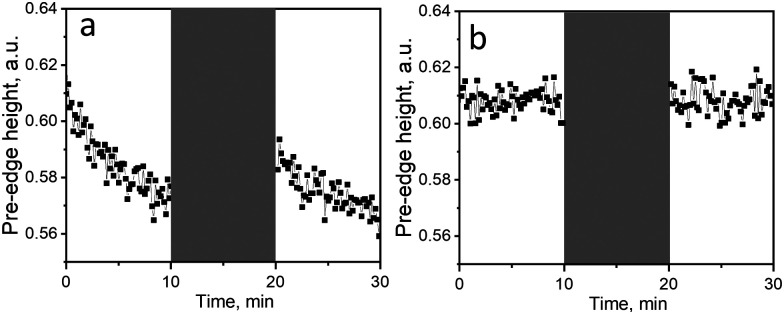
The pre-edge height changes during the beam switching experiment performed at (a) 50 and (b) 160 °C. Grey zones illustrate the periods when the X-ray beam was switched off. The feed composition was 1.6 vol% EtOH and 6.4 vol% O_2_ in He; the beam spot size was 500 × 400 μm^2^.

We also confirmed that the observed vanadium reduction is beam-induced by an X-ray beam switch-off experiment, which can be applied directly during a XAS beamtime at a synchrotron and does not require additional spectroscopies. Before the experiment, the catalyst was kept in the ethanol–oxygen mixture at 160 °C (in the absence of a beam) to reach steady-state working conditions; at the end, the temperature was adjusted to 50 or kept at 160 °C. Subsequently, we started to record the time-resolved V K-edge XAS spectra under the same reaction conditions, while periodically switching on and off the X-ray beam. The values of the V pre-edge height from such experiments at 50 and 160 °C are shown in Fig. 4; the corresponding changes in the V K-edge position are reported in Fig. S7 (ESI†). The obtained trends indicate vanadium reduction in the presence of the X-ray beam at 50 °C (Fig. 4a), without appreciable catalytic activity. In the absence of the X-ray beam, vanadium is re-oxidized, presumably by oxygen present in the feed. The experiment performed at 160 °C (temperature, at which the catalyst is active in ethanol ODH, Fig. 4b) did not reveal any redox transformations upon exposure to the X-ray beam; the speciation of vanadium before and after X-ray beam switching experiments was identical. Possible beam damage at 160 °C, where the thermocatalytic process of ethanol ODH takes place, was ruled out in our previous work,^36^ where we performed oxygen cut-off experiments (switching between EtOH + O_2_ and EtOH feeds) on the same catalyst with different beam sizes (ranging from 150 × 150 μm^2^ to 500 × 400 μm^2^). The reversible changes in the pre-edge intensity profiles, related to reversible oxidation and reduction of VO_*x*_ species, were independent of the X-ray brilliance (Fig. S8, ESI†). At the same time, the redox activity of VO_*x*_ species was accelerated by temperature,^36^ which is typical for thermocatalytic processes. This confirms that the X-ray beam used in our experiments affects the vanadium oxidation state only at low temperatures, in the absence of the thermocatalytic process. At higher temperatures, X-ray-induced reduction of vanadium was not detected, which must be associated with much faster chemical processes, taking place under these conditions.

### Influence of the feed composition

**Fig. 5:**
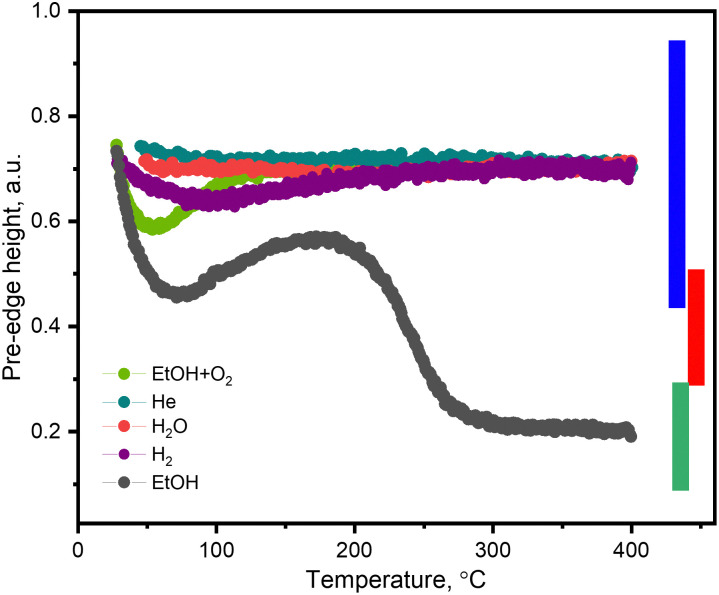
The pre-edge height temperature profiles of the 5 wt% V_2_O_5_/15 wt% TiO_2_/SiO_2_ catalyst during TPE-XAS experiments in ethanol–oxygen (1.6 vol% EtOH, 6.4 vol% O_2_ in He, heating rate 5 °C min^−1^, light-green curve), helium (purity 99.997%, heating rate 5 °C min^−1^, green curve), water (0.12 vol% in He, red curve), hydrogen (55 vol%, heating rate 10 °C min^−1^, purple curve) and ethanol (1.6 vol% EtOH in He, heating rate 5 °C min^−1^, black curve). The beam size is 200 × 400 μm^2^.

To understand the nature and conditions of X-ray-induced vanadium reduction, we followed the vanadium state in the 5 wt% V_2_O_5_/15 wt% TiO_2_/SiO_2_ catalyst by V K-edge XAS during TPE experiments in different feed compositions. To identify whether the X-ray beam alone can induce changes in the state of vanadium, we heated the fully oxidized catalyst in a flow of inert helium. In another XAS study, it was reported that copper in Cu-containing zeolites can undergo photoreduction in helium feed during XAS measurements.^27^ An XPS investigation previously showed that supported V_2_O_5_ can undergo reduction to V_2_O_4_ and V_2_O_3_ under X-ray irradiation due to the loss of oxygen.^41–43^ The intensity of the pre-edge and the edge position (Fig. 5 and Fig. S9, ESI,† green curves) of the V K-edge XAS spectra measured during TPE in helium did not change significantly over the whole temperature interval (30–400 °C). This suggests that the X-ray beam alone does not cause vanadium reduction but needs a feed that could act as an electron donor.

There are several examples in the literature where water as a solvent was identified as a source of electrons for metal reduction during XAS investigations.^21–23^ In this case, the electrons can be generated in the water radiolysis process:
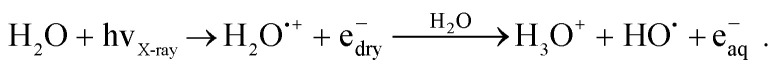


The thus formed electrons may reduce metal species, whereas radicals and ions may recombine to produce H_2_O_2_ and H_2_.^21,71^ Even though our feed does not contain a large excess of water during *in situ*/*operando* XAS experiments, traces of water can still be present in the feed (from the gas bottle and as a product of catalytic ethanol oxidation). To verify the effect of water, we exposed the catalyst to a water-containing feed (0.12 vol% H_2_O in He, which corresponds to a water concentration produced over the catalyst at *ca.* 10% ethanol conversion) during an XAS-TPE experiment. The pre-edge intensity and the half-height edge position profiles are shown in Fig. 5 and Fig. S9, ESI† (red curves). No considerable changes in the pre-edge intensity or the edge position were observed, suggesting that in the presence of water, no X-ray-induced beam reduction takes place.

Another possible source of electrons in the feed, required for vanadium reduction, is ethanol. To see the effect of the X-ray beam in the whole temperature interval, we performed ethanol XAS-TPE. As soon as ethanol reaches the catalyst, vanadium undergoes reduction, which is reflected by a decrease of the pre-edge height and a shift in the edge position (Fig. 5 and Fig. S9, ESI,† respectively, black curve). Upon heating, vanadium continues to reduce, reaching its maximum reduction at *ca.* 75 °C. A further increase in temperature leads to partial re-oxidation of vanadium, which we associate with oxygen traces present in the feed. We estimated the concentration of oxygen traces in the operando cell of *ca.* 0.02 vol% under our working conditions based on the residual (background) transformation of ethanol into acetaldehyde over a 5 wt% V_2_O_5_/15 wt% TiO_2_/SiO_2_ catalyst of 1 × 10^−6^ mol_AcH_ min^−1^ measured in an ethanol–helium flow of 50 mL min^−1^ at 160–210 °C using 15–20 mg of the catalyst and considering a complete transformation of oxygen traces into acetaldehyde^36^ At 120 °C, catalytic ethanol oxidation starts (Fig. 1b), which competes with re-oxidation, and starting from 190 °C, chemical vanadium reduction by ethanol prevails over all other processes. Full vanadium reduction is reached at 350 °C. Thus, by monitoring the pre-edge height measured during ethanol TPE, we can observe both reduction processes: X-ray beam-induced reduction (30–100 °C) and chemical reduction (150–350 °C).

Next, we tested whether in the presence of another reducing agent, *i.e.* hydrogen, the catalyst may undergo X-ray-induced reduction. The pre-edge intensity and the edge position profiles during hydrogen TPE XAS are shown in Fig. 5 and Fig. S9, ESI† (purple curves). Partial vanadium reduction was observed in the 50–180 °C temperature interval. Also in this case, the traces of oxygen in the feed must have re-oxidized the vanadium species at higher temperatures; starting from 200 °C the oxidation state of vanadium species returned to the initial fully oxidized one. At 400 °C hydrogen was not able to reduce vanadium, in agreement with the observation that reduction takes place at *ca.* 530 °C.^44^ Furthermore, the extent of vanadium reduction in the hydrogen TPE was significantly lower than that in ethanol TPE. This correlates to the generally lower ability of hydrogen to reduce this catalyst. To summarize, X-ray beam-induced vanadium reduction occurs only in the presence of reducing agents, such as ethanol or hydrogen.

We additionally tested whether titania (being photoactive^72^) induces the observed photochemical reduction of vanadium. For this, we performed ethanol TPE-XAS on a titania-free 8 wt% V_2_O_5_/SiO_2_ sample. The V K-edge XAS also revealed a very similar vanadium reduction for the titania-free catalyst in the low-temperature interval (see ESI,† for additional figures and discussion, Section 3.3). This showed that photochemical reduction of vanadium is not related to or promoted by the photoactive titania support.

At low temperatures, no products of ethanol oxidation were detected in the outlet, which may be related to the small amount of the catalyst exposed to the X-rays or to the low sensitivity of the IR-spectrometer used for product analysis. Furthermore, the products could be strongly adsorbed on the catalyst surface.

### The effect of beam brilliance and intensity

The next goal of our investigation was to identify whether X-ray-induced vanadium reduction can be avoided or diminished by defocusing the beam and/or by applying filters. This strategy was successfully used to avoid copper photoreduction observed during catalytic methane oxidation over Cu-containing zeolites.^27^ The majority of experiments discussed so far (unless stated otherwise) were performed using an X-ray beam with a spot size at the sample position equal to 200 × 400 μm^2^ (this corresponds to a brilliance of *ca.* 1.5 × 10^12^ ph mm^−2^ s^−1^). To diminish the rate of the X-ray-induced vanadium reduction, we defocused the incident X-ray beam to a size of 500 × 400 μm^2^. This reduced the brilliance by a factor of 2.5 while preserving the total flux, and consequently, the quality of the XAS spectra. In Fig. S10 (ESI†), we plotted the pre-edge height profiles obtained during the ethanol TPE experiment with the use of focused and defocused beams. One can see that the extent of vanadium reduction at a low temperature is significantly decreased; however, it is still present. The X-ray beam switching experiments, shown in Fig. 5, were also performed with the use of the defocused beam (500 × 400 μm^2^), and as we discussed before, vanadium reduction was observed upon X-ray irradiation at 50 °C.

**Fig. 6:**
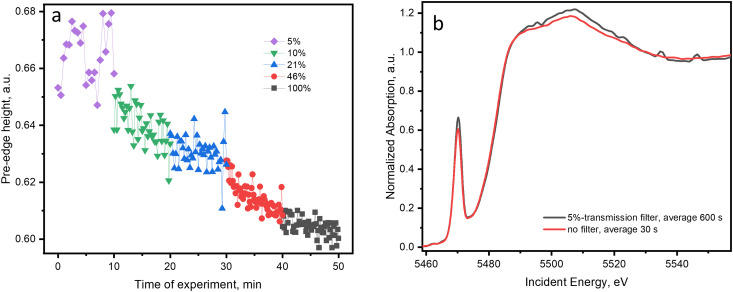
(a) Pre-edge height of the V K-edge spectra of the 5 wt% V_2_O_5_/15 wt% TiO_2_/SiO_2_ catalyst measured in ethanol–oxygen feed (1.6 vol% EtOH, 6.4 vol% O_2_ in He) at 50 °C with the use of different Al-filters. The percentage numbers indicate the intensity of the beam relative to the beam without filter (intensity of the transmitted beam). (b) Comparison of the quality of a V K-edge XANES spectrum measured with the use of a 5%-transmission filter (averaged within 600 s) and without filter (averaged within 30 s); the beam spot size was 500 × 400 μm^2^.

We attempted to further decrease the effect of the X-ray beam and find conditions that would allow the investigation of the supported VO_*x*_ catalysts at low temperature in the presence of a reducing feed. For this, the intensity of the defocused incident beam (500 × 400 μm^2^) was additionally decreased by a factor of 2–20 by applying aluminum filters of different thicknesses. Before the experiment, the 5 wt% V_2_O_5_/15 wt% TiO_2_/SiO_2_ catalyst was exposed to the ethanol–oxygen feed in the absence of the X-ray beam for 20 min at 50 °C. This allowed to reach equilibrium. Afterward, the thickest Al-filter (which absorbs 95% of the incident beam) was applied and V K-edge XANES was recorded for 10 min (1200 scans). The same procedure was followed by filters with smaller thicknesses. In Fig. 6, we plotted the pre-edge height values with the use of different filter thicknesses (the V K edge XAS spectra are in Fig. S11, ESI†). The evolution of the pre-edge height in Fig. 6a suggests that vanadium reduction in the low-temperature regime occurred even with a reduced beam intensity. Only the measurement performed with the use of 5% of the full beam intensity (corresponding to a beam brilliance of 3 × 10^10^ ph mm^−2^ s^−1^) did not reveal vanadium reduction. However, by applying such filters, we decrease the total flux (Table S1, ESI†), which leads to a significant decrease in the spectral quality. This can be partially compensated by a longer acquisition time (Fig. 6b). However, despite the 20 times higher number of averaged scans, the resulting spectrum acquired at a lower X-ray intensity loses quality, evidenced by the monochromator glitches appearing in the interval of 5530–5550 eV, which is caused by the different nonlinear responses of the detectors used (*i.e.* the ionization chamber *vs.* the PIPS detector).

Thus, X-ray-induced vanadium photochemical reduction at low temperatures can be significantly diminished by using a defocused X-ray beam, which does not affect the quality of the spectra. The use of filters may further reduce the effect of X-ray irradiation which is necessary when studying rates of reduction and oxidation; however, poor spectral quality may become a limiting factor in efficient time-resolved V K-edge XAS acquisition.

## Conclusions

During V K-edge XAS studies at a third-generation synchrotron facility, we detected X-ray-induced vanadium photoreduction, which strongly affected the chemical speciation of the supported VO_*x*_ species below the temperature of thermocatalytic ethanol oxidation. The variation of the gas feed composition revealed that the photoreduction occurs only in the presence of a reducing agent (ethanol, hydrogen, or ethanol–oxygen mixture) in the gas feed and is particularly evident below 100 °C. The X-ray-induced vanadium photoreduction was also observed on a titania-free 8 wt% V_2_O_5_/SiO_2_ catalyst, showing that photoreduction of vanadium cannot be explained by the photoactive titania. Defocusing the incident beam significantly reduces the photoreduction effect without losing the spectral quality of the XAS data. The use of X-ray filters can further reduce photoreduction; however, it deteriorates the spectral quality, which ultimately can become a limiting factor for probing the vanadium speciation by time-resolved XAS. To exclude beam damage under specific *in situ/operando* conditions, we suggest performing X-ray beam switching (on and off) tests at different beam intensities.

## Author contributions

A. Z. and O. S. planned the research. A. Z., A. H. C., and O. V. S performed XAS experiments and contributed to the data analysis. A. Z. under the guidance of D. Ferri performed the UV-Vis experiments and analyzed the results. A. Z. drafted the manuscript. All authors contributed to the scientific discussion and provided corrections to the manuscript.

## Conflicts of interest

There are no conflicts to declare.

## Supplementary Material

CP-024-D2CP02721F-s001
