# Supplementary material for: Beware of beam damage under reaction conditions: X-ray induced photochemical reduction of supported VOx catalysts during in situ XAS experiments
Source: Phys Chem Chem Phys. 2022 Aug 25;24(36):21916–26. doi: 10.1039/d2cp02721f (PMC9641748; doi:10.1039/d2cp02721f)
Supplement: CP-024-D2CP02721F-s001 [file CP-024-D2CP02721F-s001.pdf]

Electronic Supplementary Material (ESI) for Physical Chemistry Chemical Physics.
This journal is © the Owner Societies 2022

Supplementary Information:

Beware of beam damage under reaction conditions: X-ray induced photochemical reduction of supported VO_x catalysts during *in situ* XAS experiments

Anna Zabilska[1,2], Adam H. Clark[1], Davide Ferri[1], Maarten Nachtegaal[1], Oliver Kröcher[1,2], Olga V. Safonova[1]*

[1]Paul Scherrer Institute, 5232 Villigen, Switzerland

[2]École Polytechnique Fédérale de Lausanne, 1015 Lausanne, Switzerland

Contents

1. Supplementary methods 3
 1.1. *In situ* DR-Vis measurements 3
 1.2. Experiments with aluminum filters 3
 1.3. V pre-edge analysis 4
 1.4. Quantitate analysis of the products by IR spectrometry 5
2. Supplementary Figures and Tables 6
3. Supplementary Results and Discussion 11
 3.1. V K-edge descriptors 11
 3.2. DR UV-Vis experiments 12
 3.3. Effect of the support 14
4. Supplementary References 17

1. Supplementary methods

1.1. *In situ* DR-Vis measurements

The *in situ* diffuse reflectance UV-Visible (DR UV-Vis) spectroscopy study was performed on an Agilent Carry 4000 spectrometer equipped with a Praying Mantis mirror unit (Harrick). The commercial Harrick cell was equipped with a homemade CaF_2 window (d = 25 mm, thickness 2 mm; Crystran) in place of the commercial dome. The catalyst sample (ca. 25 mg) was placed in the sample cup. The pre-treatment and temperature-programmed experiments were performed following the protocol described for the V K-edge XAS experiments. Spectra were recorded only in 350 – 800 nm interval in order to exclude the signal saturation at 250 – 350 nm (Figure S6) and to enhance the signal intensity in the *d-d* transition region.

1.2. Experiments with aluminum filters

To reduce the intensity of the incident beam, aluminum filters with different thickness were utilized. On the Figure S1 b, the thickness of Al-foil which provides *e* times diminished beam versus incident energy is shown. On Figure S1 c, the thickness of used filters and corresponding intensities are marked with “x”.

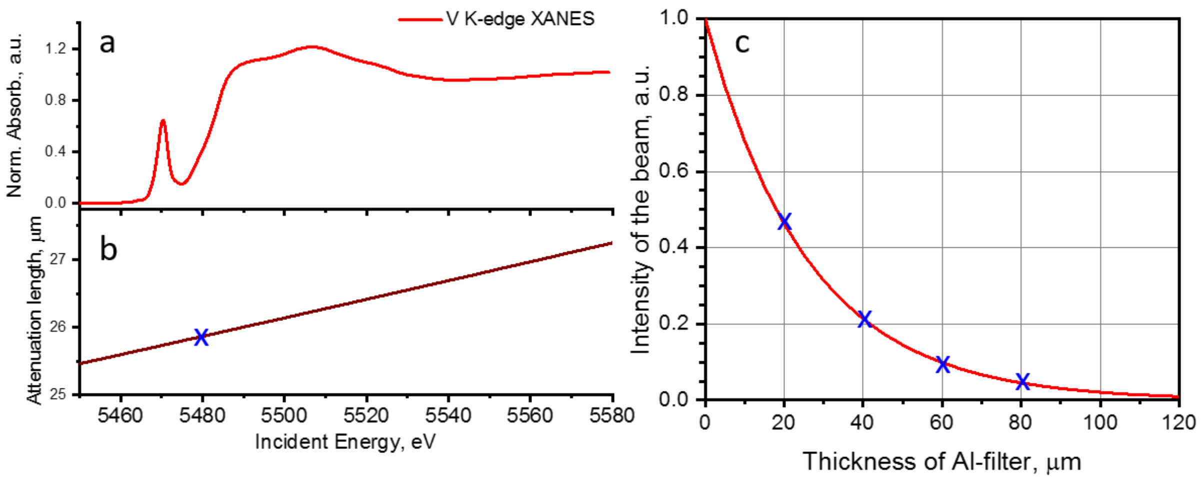

Figure S1. (a) V K-edge spectrum of the catalyst (shown for energy scale), (b) attenuation length of Al-foil (the depth into material where the intensity of the incident X-ray beam falls in e times) versus incident energy value. The value used for further calculation of the beam intensity is marked with ‘x’. (c) Intensity of the incident beam versus Al-filter length, the values of used in this work filters are marked with ‘x’.

Table S1. The X-ray beam characteristics used in this work.

Beam size, μm^2	Al-Filter thickness, μm	Intensity of the beam relative to the beam without filter (transmission intensity, a.u.	Total flux, photons · s^{-1}	Brilliance, photons · mm^{-2} · s^{-1}
150 x 150	No filter	1	1.2×10^{11}	5.3×10^{12}
300 x 200	No filter	1	1.2×10^{11}	2×10^{12}
300 x 400	No filter	1	1.2×10^{11}	1×10^{12}
500 x 200	No filter	1	1.2×10^{11}	1.2×10^{12}
200 x 400	No filter	1	1.2×10^{11}	1.5×10^{12}
500 x 400	No filter	1	1.2×10^{11}	6×10^{11}
	20	0.46	5.5×10^{11}	2.8×10^{11}
	40	0.21	2.5×10^{11}	1.3×10^{11}
	60	0.1	1.2×10^{10}	6×10^{10}
	80	0.05	6×10^{9}	3×10^{10}

1.3. V pre-edge analysis

For more accurate calculation of the pre-edge area and pre-edge center of mass, cumulative distribution function (CDF, S1) was used as a baseline to correct the edge rising (Figure S2). The edge jump was fitted with CDF with the use of least square method (implemented on the base of Python). The resulting peak was integrated. In the case if an edge arise contained an additional shoulder (B-peak in the Figure S3), the resulting after subtraction peaks were fitted with multiple pseudo-Voight functions to identify and subtract the contribution of the shoulder (Figure S3 c). The detailed fits of all used in this work references could be found in [1].

$$f_{CDF} = \frac{1}{2}\left[1 + erf\left(\frac{x-\mu}{\sigma\sqrt{2}}\right)\right] \quad \text{(S1)}$$

Where x is the energy scale, μ is a fitted parameter, which defines the x position of f_{CDF} and σ is a fitted parameter, which defines the slope of f_{CDF}.

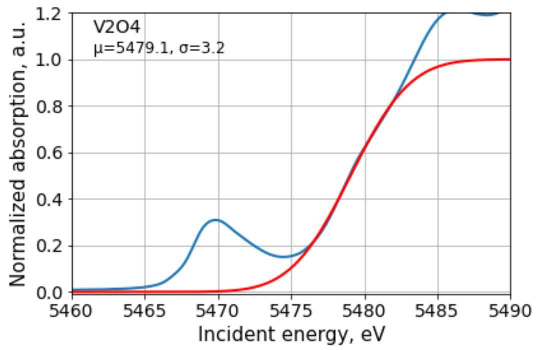

Figure S2. V K-edge spectrum of V_2O_4 and fitted CDF subtracted as as a baseline prior to integration.

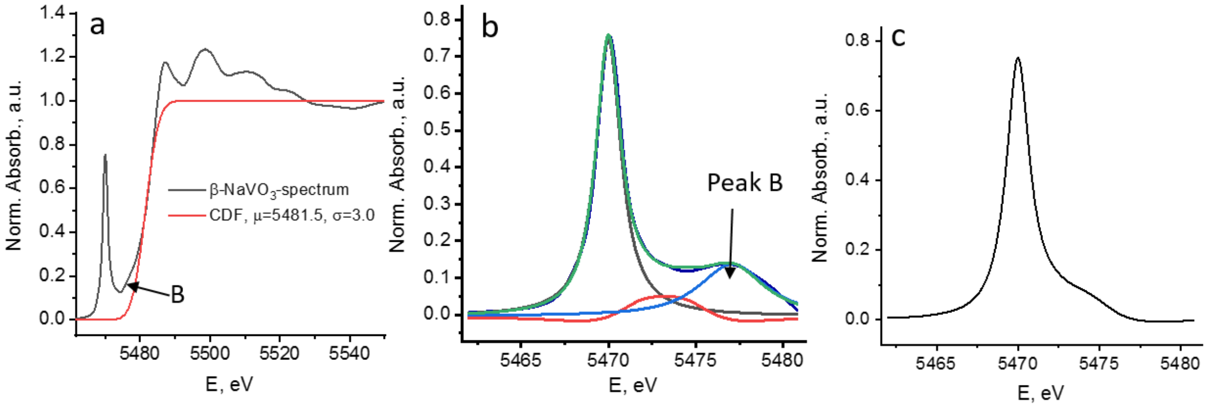

Figure S3. The procedure for the pre-edge area calculation when the edge arise contained an additional shoulder (B). (a) Fit edge with a cumulative distribution function; (b) resulting curve fitted with multiple pseudo-Voight functions; (c) subtraction of the additional pre-edge peak (peak B).

1.4. Quantitate analysis of the products by IR spectrometry

The spectra of ethanol, acetaldehyde and other possible products are shown in Figure S3. For the quantitate analysis of ethanol and acetaldehyde, the bands in the 1294 – 1181 cm^{-1} (ethanol) and 1853 – 1658 cm^{-1} spectral ranges (acetaldehyde) were extracted and analyzed with the use of ALS-MCR. For quantitative calibration, a series of spectra containing pure ethanol or acetaldehyde in different known concentrations were included to the analysis. Two constraints, non-negativity of the component fraction and sum of all components lower than 1, were applied.

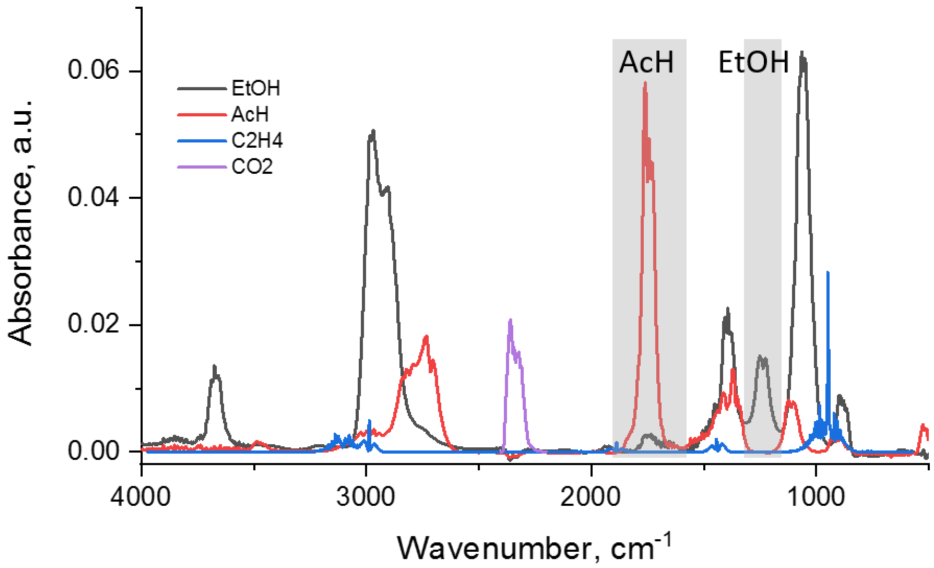

Figure S4. IR spectra of pure ethanol, acetaldehyde, ethene and carbon dioxide. The bands used for quantitative analysis of acetaldehyde (AcH) and ethanol (EtOH) are highlighted with grey color.

2. Supplementary Figures and Tables

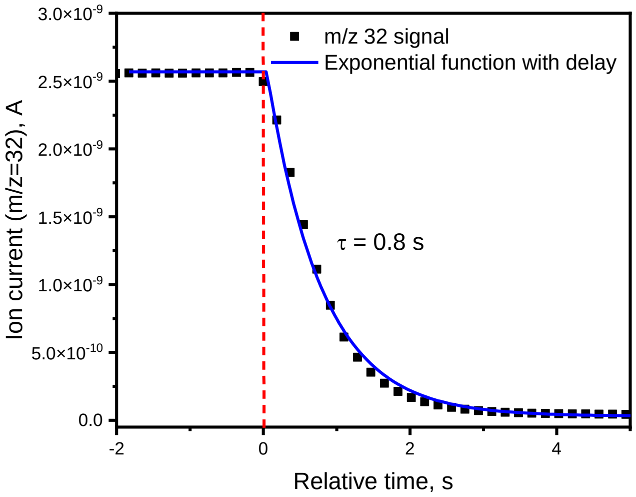

Figure S5. Rate of gas exchange in the *operando* cell measured by mass spectrometry. Oxygen was replaced by helium at the time 0 s. Total flow 50 mL/min. This experiment was done with the use of the mass-spectrometer OmniStar GSD 320, Pfeiffer

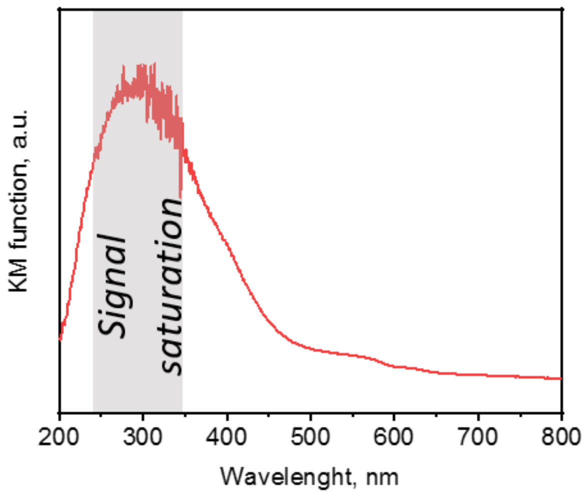

Figure S6. UV-Vis spectrum of 5 wt% V_2O_5/15 wt% TiO_2/ SiO_2 catalyst measured in the 200 – 400 nm range. We neglected the signal saturation below 340 nm to increase the signal intensity in d-d transition region.

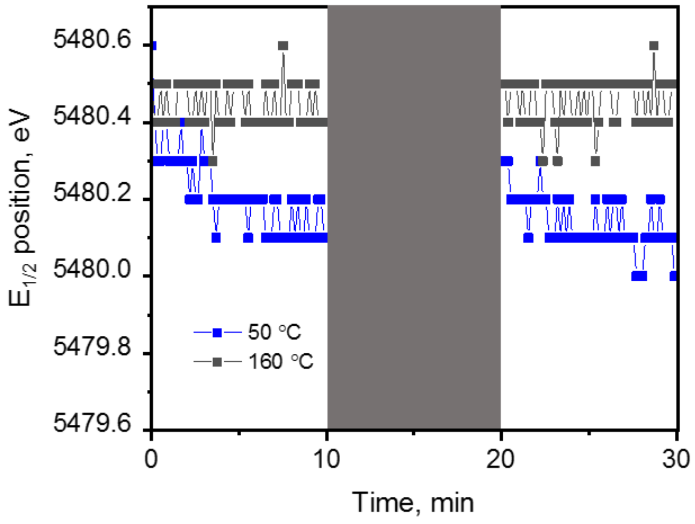

Figure S7. The half-height edge position profile in the beam switch-off experiment performed at 50 and 160 °C. The shaded area illustrates the time when X-ray beam was switched off. Feed composition: 1.6 vol% EtOH, 6.4 vol% O_2 in He.

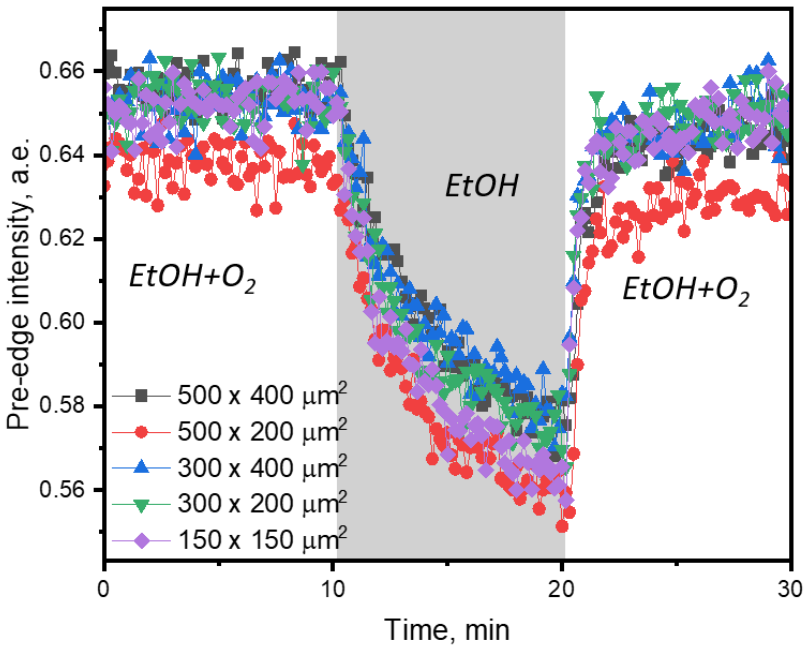

Figure S8. The V K-edge pre-edge intensity of the 5% V_2O_5/15% TiO_2/ SiO_2 catalyst measured in oxygen cut-off experiments at 160 °C (1.6 vol% EtOH+ 6.4 vol% O_2 in He was replaced by 1.6 vol% EtOH in He) performed with the use of differently sized X-ray beams (the corresponding values of beam brilliance can be found in Table S1). For more details refer to [1]. At 160 °C, the thermocatalytic processes (oxidation and reduction of vanadium involved in catalytic transformation of ethanol into acetaldehyde) become significantly faster than the photocatalytic reduction of V^{5+}. For this reason, the observed trends are rather independent on the X-ray brilliance.

.

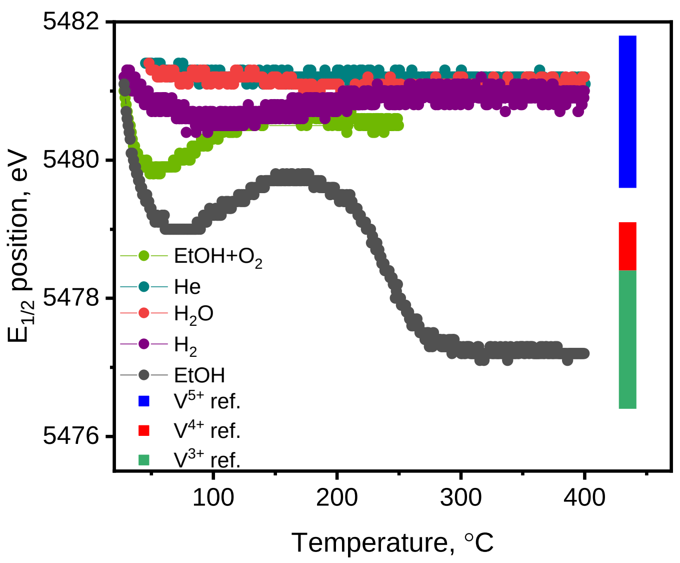

Figure S9. The half-height edge position during TPE in ethanol-oxygen (1.6 vol% EtOH, 6.4 vol% O_2 in He, 5 °C/min, light-green curve), helium (purity 99.997%, 5 °C/min, green curve), water (0.12 vol% in He, red curve), hydrogen (55 vol%, 10 °C/min, purple curve) and ethanol (1.6 vol% EtOH) flows, 5 °C/min, black curve). The vanadium references are represented by different bars (details in Figure 12).

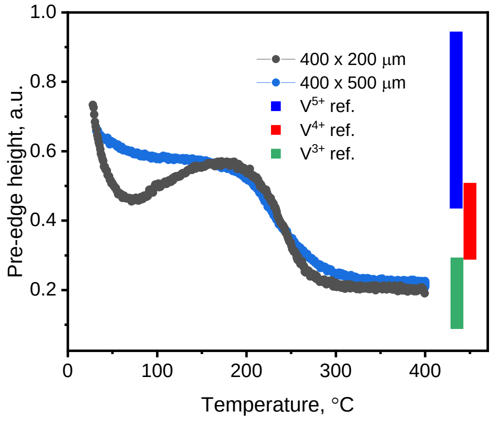

Figure S10. The pre-edge height temperature profile during ethanol TPE measured using an X-ray beam size of 200 x 400 μm^2 and 500 x 400 μm^2 at the sample position. Colored bars are used to show the pre-edge height of vanadium references.

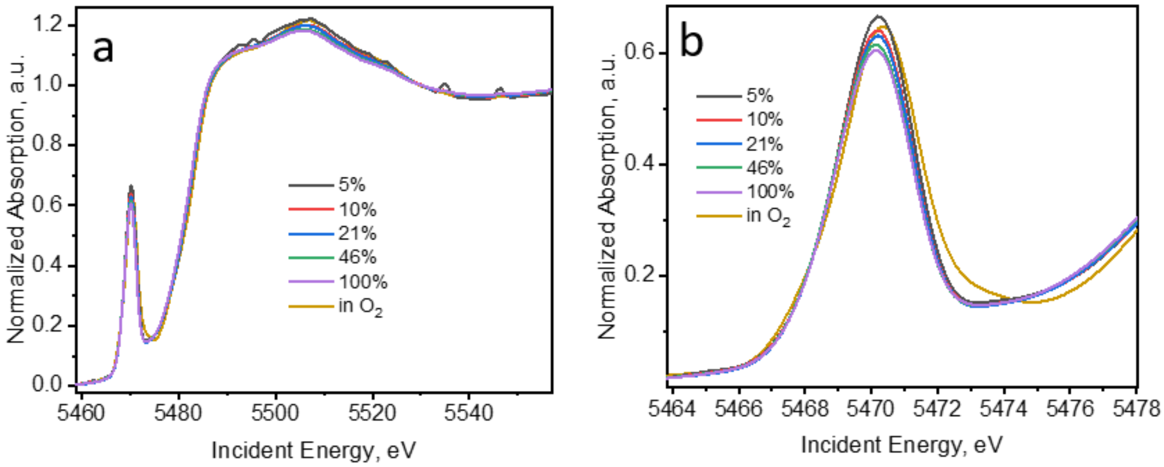

Figure S11. Full V K-edge XANES (a) and pre-edge interval (b) of the 5 wt% V_2O_5/15 wt% TiO_2/SiO_2 catalyst measured in ethanol-oxygen feed (1.6 vol% EtOH, 6.4 vol% O_2 in He) at 50 °C with the use of different filters. Spectra are the result of average over 10 min (1200 scans). The spectrum of the catalyst in oxygen flow (prior to ethanol-oxygen exposure) is shown in yellow color. The spectra were averaged by 30 s (for 5%-transmission filter), 15 s (for 10%- and 21%-filters) and 10 s (for 46%-filter and no filter measurement). Percentage numbers indicate the intensity of the beam relative to the beam without filter (intensity of transmitted beam).

3. Supplementary Results and Discussion

3.1. V K-edge descriptors

Figure S12 shows the V K-edge XANES descriptors, namely the edge position (determined as the half-height edge position), the pre-edge height, the area under the pre-edge peak, and the pre-edge center of mass position. With different blue dots, we indicate V^{5+} species; different shades correspond to different coordination numbers. V^{4+} and V^{3+} species are depicted in red and green, respectively. In agreement with the previous studies, the edge position and the pre-edge intensity (height and area) generally increase in the order V^{3+} - V^{4+} - V^{5+}. The edge position does not strongly depend on different geometries of V^{5+}, whereas the pre-edge height and the pre-edge area change as a function of vanadium coordination number. The position of the pre-edge centroid (center of mass) is noted as the least sensitive spectral signature; the measured references do not demonstrate any trend showing a wide overlapping variation for the V^{5+} and V^{4+} compounds. Although the area under the pre-edge peak (Figure S12) is a more precise descriptor of the oxidation state than the pre-edge height and allows to distinguish 5/6-coordinated V^{5+} from V^{4+}, its determination requires user-controlled background subtraction and peak fitting, which makes analysis more time-consuming. Thus, in this work, for the rapid qualitative analysis of the oxidation state of vanadium, we decided to use two descriptors: the pre-edge height and the edge position.

N-r	Compound	Ox. State	Coor. N-r
1	NH_4VO_3	+5	4
2	KVO_3	+5	4
3	α-$NaVO_3$	+5	4
4	VO(O-*i*-Pr)$_3$	+5	4
5	VO(OTBOS)$_3$	+5	4
6	K_3VO_4	+5	4
7	$BiVO_4$	+5	4
8	V_2O_5	+5	5
9	β-$NaVO_3$	+5	5
10	NaV_6O_{15}	~+5	5
11	$Na_{1.164}V_2O_5$	~+5	5.33
12	$(NH_4)_6V_{10}O_{28}\cdot 6H_2O$	+5	6
13	$Na_6V_{10}O_{28}\cdot 18H_2O$	+5	5.67
14	V(OTBOS)$_4$	+4	4
15	VO(acac)$_2$	+4	5
16	V_2O_4	+4	6
17	$VOSO_4$	+4	6
18	V(OTBOS)$_3$OPPh$_3$	+3	4
19	V(OTBOS)$_3$thf$_2$	+3	5
20	V_2O_3	+3	6
21	V(acac)$_3$	+3	6

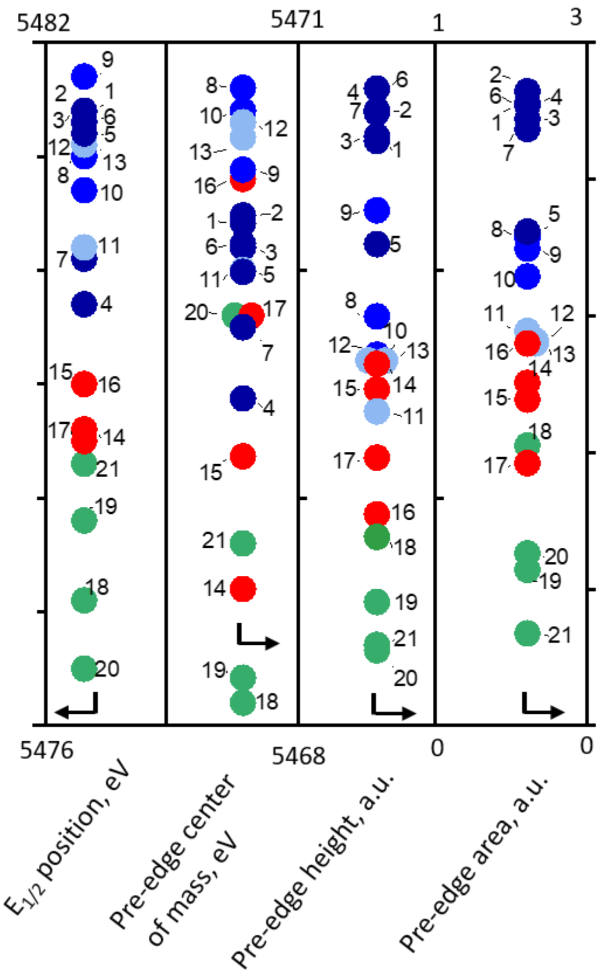

Figure S12. List of vanadium-containing references and V K-edge XAS descriptors: the half-height edge step position, the pre-edge height, the area under the pre-edge peak, and the pre-edge center of mass position. Different colors represent different oxidation states of vanadium. Different shades of blue dots show different coordination numbers of V^{5+}.

3.2. DR UV-Vis experiments

Electronic spectroscopy is also sensitive to oxidation state changes in this type of catalysts [2]. Therefore, we performed similar experiments using diffuse reflectance UV-Vis spectroscopy (DR UV-Vis; Figure S13). First, we performed ethanol TPE (1.6 vol% EtOH in He) and ethanol-oxygen TPE (1.6 vol% EtOH, 6.4 vol% O_2 in He) experiments in the temperature range 50-400 ^oC as shown in Figure S13 a and b, respectively. The spectra recorded in ethanol feed demonstrated a significant increase in the spectral range between 400 and 800 nm centered at

around 558 nm that is characteristic of d-d transitions of V^{4+}/V^{3+} ions [3]. In the ethanol-oxygen feed, the change was much less evident.

The *in situ* DR UV-Vis measurement in ethanol feed at 50 °C is shown in Figure S13 c together with the reference spectrum (red) of the catalyst reduced in ethanol feed (1.6 vol% EtOH in He) at 400 °C. Contrary to the TPE experiment and to the V K-edge XANES observations, only very faint changes were observed in the ethanol feed at 50 °C suggesting that changes in the oxidation state of vanadium are negligible at this temperature and under these conditions.

To follow the transformation of vanadium species in these DR UV-Vis experiments, we display in Figure S13 d the value of the Kubelka-Munk (KM) function at 558 nm measured in ethanol TPE and ethanol-feeding experiments. These experiments prove that DR UV-Vis is sensitive to the formation of reduced V^{4+}/V^{3+} species, which can be clearly identified upon heating in ethanol feed above 150 ºC. However, the changes are very small in the ethanol-feeding experiment at 50 °C. Thus, the DR UV-Vis experiments demonstrate that vanadium reduction in ethanol feed below 100 ºC occurs only during the V K-edge XAS experiment, whereas in the absence of the X-ray beam, vanadium remains mostly in V^{5+} state.

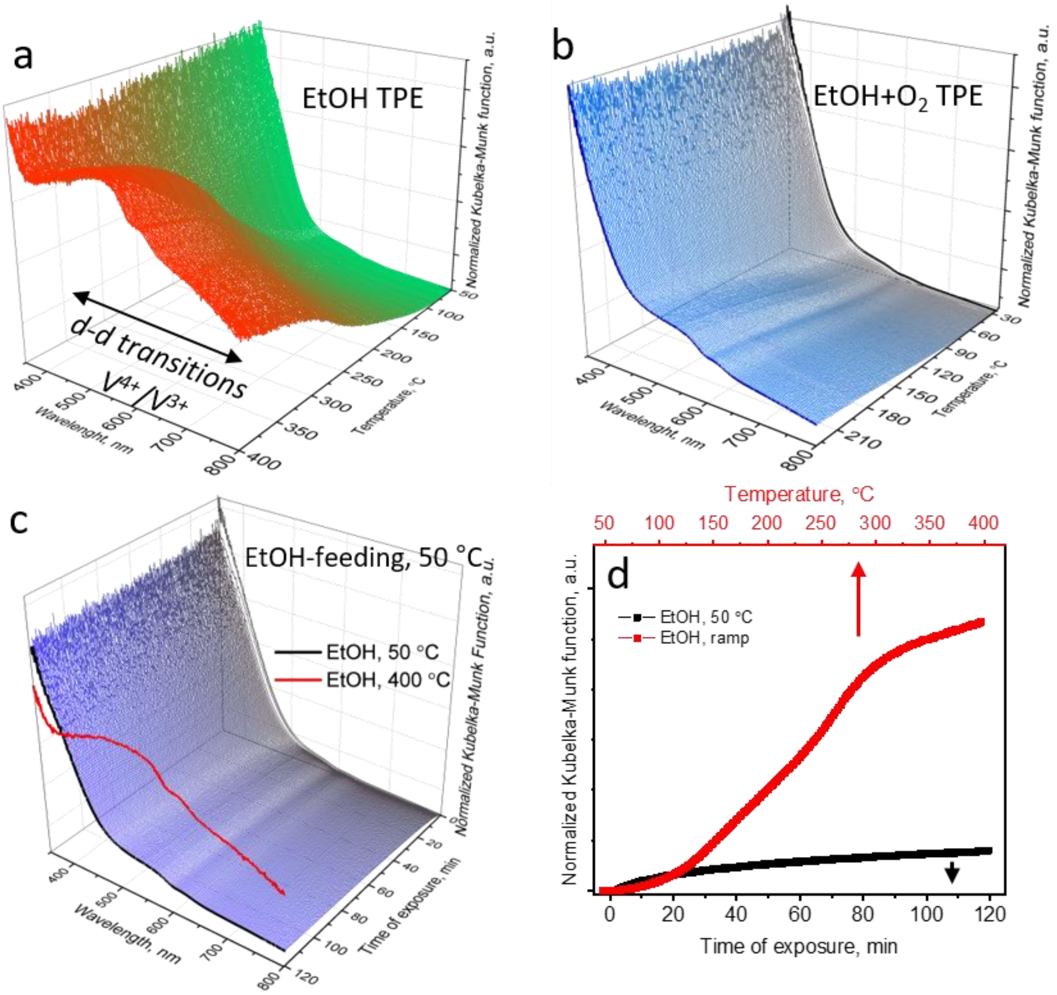

Figure S13. DR UV-Vis spectra of the 5 wt% V_2O_5/15 wt% TiO_2/SiO_2 catalyst measured during TPE in (a) ethanol (1.6 vol% EtOH in He) and (b) ethanol-oxygen feed (1.6 vol% EtOH, 6.4 vol% O_2 in He). (c) DR UV-Vis spectra obtained in the ethanol-feeding experiment at 50 °C (1.6 vol% EtOH in He). The spectrum of the catalyst reduced in ethanol (1.6 vol% EtOH in He) at 400 °C is shown in red. (d) The KM function values measured at 558 nm in ethanol-feeding experiment and during ethanol TPE (1.6 vol% EtOH in He).

3.3. Effect of the support

The bilayered 5 wt% V_2O_5/15 wt% TiO_2/SiO_2 catalyst, as was mentioned in the experimental part, serves as a model of VO_x/TiO_2 catalyst, which demonstrates outstanding catalytic activity in a number of chemical reactions. For instance, the rate of acetaldehyde production over titania-supported vanadia is ca. 2 orders of magnitude higher than over silica-supported vanadia

[1,2]. Using density functional theory (DFT), it was shown that the ability of titania to form oxygen vacancies in close proximity to surface vanadia species may facilitate vanadium reduction during catalytic reactions and, therefore, increase its activity [4–6]. Besides, titania itself is a well-known photocatalyst and prone to generate electron-hole pairs upon light exposure [7]. In this regard, we had to test, whether titania-support is facilitating X-ray beam-induced vanadium reduction. For this, we performed ethanol TPE over titania-free 8 wt% V_2O_5/SiO_2 catalyst. The activity of this catalyst in ethanol ODH is significantly lower, (Figure S14 b); the acetaldehyde production curve is shifted by ca. 90 °C in comparison to one of 5 wt% V_2O_5/ 15 wt% TiO_2/ SiO_2 catalyst. In Figure S14 a, we plotted the pre-edge height of V K-edge spectra measured during ethanol TPE over 5 wt% V_2O_5/ 15 wt% TiO_2/ SiO_2 and 8 wt% V_2O_5/SiO_2 catalysts. These pre-edge height profiles could be conventionally divided into two parts, low temperature (30-110 °C) and high temperature (110-400 °C). Whereas the vanadium reduction in the high-temperature part is related to ethanol ODH and depends on the support (Figure S14 b), the reduction in the low-temperature part is related to X-ray induced reduction and seems to be very similar on different catalysts. It suggests that photochemical process is not strongly influenced by the presence of titania. Additionally, we performed the ethanol-feeding experiment over VO_x/SiO_2 catalyst at 30 °C, the pre-edge intensity and the edge position profiles of both catalysts are shown in Figure S14 c and d. Similarly to bilayered $VO_x/TiO_2/SiO_2$ catalyst, titania-free VO_x/SiO_2 catalyst demonstrates strong vanadium reduction at 30 °C in the presence of ethanol. Moreover, the rate of vanadium reduction is faster on VOx/SiO_2 catalyst, which could be explained by partial X-ray absorption by TiO_x support in $VO_x/TiO_x/SiO_2$ catalyst. The ability of the catalyst to be reduced by X-ray beam does not correlate with the chemical activity of the catalyst in ethanol oxidation and is not facilitated by photoactive titania-support.

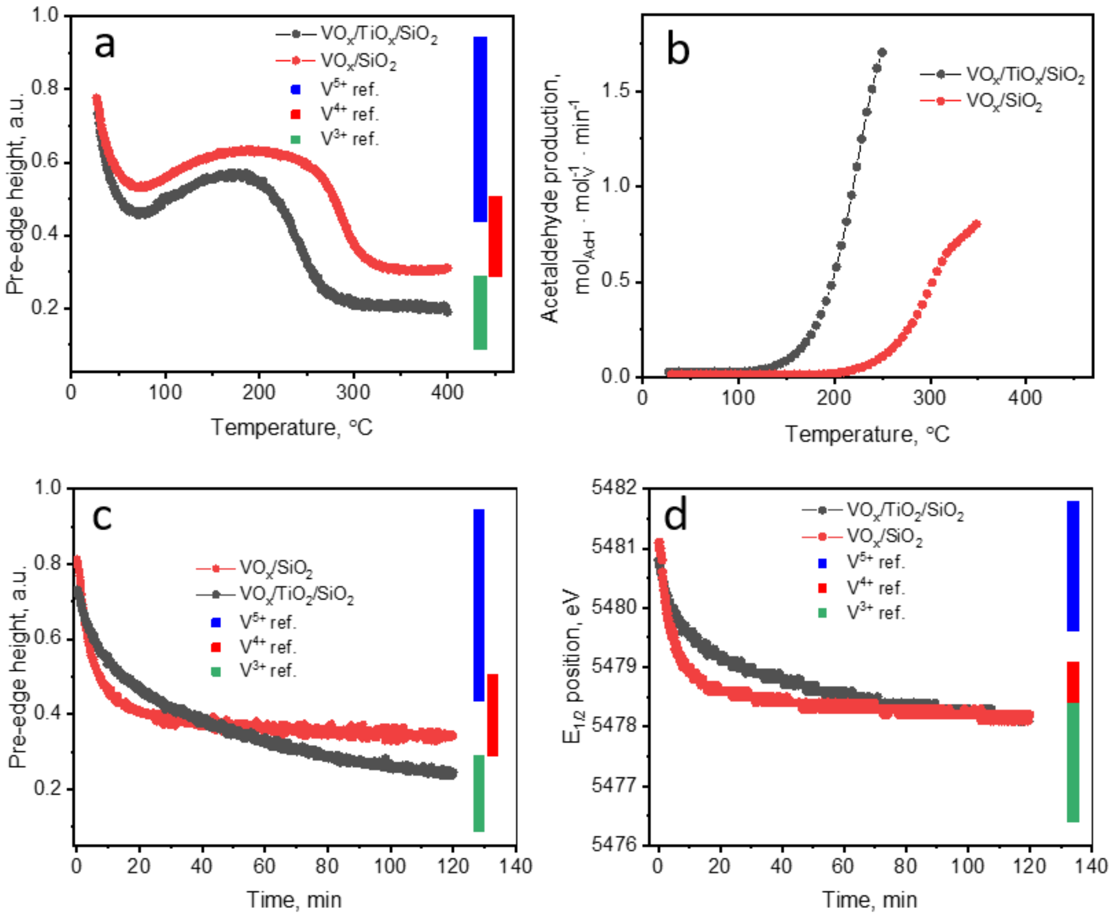

Figure S14. (a) The pre-edge height profile during ethanol TPE over the 5 wt% V_2O_5/15 wt% TiO_2/SiO_2 and 8 wt% V_2O_5/SiO_2 catalysts. (b) Corresponding acetaldehyde production rate detected in the outlet during ethanol-oxygen TPE. (c) The pre-edge height and (d) the half edge step position of the 5 wt% V_2O_5/15 wt% TiO_2/SiO_2 and 8 wt% V_2O_5/SiO_2 catalysts measured during the ethanol-feed experiments at 30 °C. Colored bars were used to show the vanadium references (details are in Figure S12). The beam size is 400 x 200 μm^2.

4. Supplementary References

[1] A. Zabilska, A.H. Clark, B.M. Moskowitz, I.E. Wachs, Y. Kakiuchi, C. Copéret, M. Nachtegaal, O. Kröcher, O. V. Safonova, JACS Au 2 (2022) 762–776.

[2] X. Gao, S.R. Bare, J.L.G. Fierro, I.E. Wachs, J. Phys. Chem. B 103 (1999) 618–629.

[3] L.J. Burcham, X. Gao, I.E. Wachs, G. Deo, Top. Catal. 11–12 (2000) 85–100.

[4] A. Goodrow, A.T. Bell, J. Phys. Chem. C 112 (2008) 13204–13214.

[5] D. Yun, Y. Wang, J.E. Herrera, ACS Catal. 8 (2018) 4681–4693.

[6] B. Beck, M. Harth, N.G. Hamilton, C. Carrero, J.J. Uhlrich, A. Trunschke, S. Shaikhutdinov, H. Schubert, H.J. Freund, R. Schlögl, J. Sauer, R. Schomäcker, J. Catal. 296 (2012) 120–131.

[7] A. Fujishima, T. Rao, D. Tryk, J. Photochem. Photobiol. C Photochem. Rev. 1 (2000) 1–21.
